# Oncoprotein HCCR-1 expression in breast cancer is well correlated with known breast cancer prognostic factors including the HER2 overexpression, p53 mutation, and ER/PR status

**DOI:** 10.1186/1471-2407-9-51

**Published:** 2009-02-11

**Authors:** Seon-Ah Ha, Youn Soo Lee, Seung Min Shin, Hyun Kee Kim, Sanghee Kim, Hong Namkoong, Hae Joo Kim, Sang Min Jung, Yu Sun Lee, Yeun Jun Chung, Sang Seol Jung, Jin Woo Kim

**Affiliations:** 1 Department of Molecular Genetic Laboratory, College of Medicine, The Catholic University of Korea, Seoul 137-040, Korea; 2 Department of Hospital pathology, College of Medicine, The Catholic University of Korea, Seoul 137-040, Korea; 3 Department of Microbiology, College of Medicine, The Catholic University of Korea, Seoul 137-040, Korea; 4 Department of Surgery, College of Medicine, The Catholic University of Korea, Seoul 137-040, Korea; 5 Department of Obstetrics and Gynecology, College of Medicine, The Catholic University of Korea, Seoul 137-040, Korea

## Abstract

**Background:**

Oncoprotein HCCR-1 functions as a negative regulator of the p53 and contributes breast tumorigenesis. The serum HCCR-1 assay is useful in diagnosing breast cancer and mice transgenic for HCCR developed breast cancers. But it is unknown how *HCCR-1 *contributes to human breast tumorigenesis.

**Methods:**

Oncogene HCCR-1 expression levels were determined in normal breast tissues, breast cancer tissues and cancer cell lines. We examined whether HCCR-1 protein expression in breast cancer is related to different biological characteristics, including ER, PR, p53 genotype, and HER2 status in 104 primary breast cancer tissues using immunohistochemical analyses.

**Results:**

HCCR-1 was upregulated in breast cancer cells and tissues compared with normal breast tissues. In this study, overexpression of HCCR-1 was well correlated with known breast cancer prognostic markers including the presence of steroid receptors (ER and PR), p53 mutation and high HER2 overexpression. HCCR-1 was not detected in the ER-negative, PR-negative, p53 negative and low HER2 breast cancer tissues. These data indicate that the level of HCCR-1 in breast cancer tissues is relatively well correlated with known breast cancer factors, including the HER2 overexpression, p53 mutation, and ER/PR status.

**Conclusion:**

Determination of HCCR-1 levels as options for HER2 testing is promising although it needs further evaluation.

## Introduction

Cells expressing HCCR-1 are tumorigenic in nude mice [1]. The functional role of this oncogene in tumorigenesis is manifested as a negative regulator of the p53 tumor suppressor [1]. In the previous study, we investigated HCCR-1 protein expression in breast cancer and the possibility of using HCCR-1 as a useful biomarker for human breast cancer [2]. We also examined whether HCCR-1 protein expression in breast cancer is related to different biological characteristics including ER, PR, p53 genotype, and the HER2 status. Northern and Western blot analyses and immunohistochemical studies indicate that the HCCR-1 mRNA and protein are overexpressed in breast cancer tissues compared to the normal breast tissues [1,2]. Serological studies revealed an 86.8% sensitivity for HCCR-1 in breast cancer, which was higher than 21.0% for CA15-3 [2,3]. The HCCR-1 assay has an advantage over CA15-3 in diagnosing breast cancer. These results indicate that HCCR-1 is an oncoprotein that is related to breast cancer development [1,2].

Overexpression of human epidermal growth factor receptor type 2 (HER2, also referred to as HER2/*neu *or ErbB-2), a 185-kD receptor first described more than two decades ago [4], occurs in 20 to 30% of invasive breast cancers. In general, patients with breast-cancer cells that overexpress this receptor or that have a high copy number of its gene have decreased overall survival and may have differential responses to a variety of chemotherapeutic and hormonal agents [5,6]. Thus, strategies to target HER2 appear to be important in treating breast cancer [7]. HER2 signaling promotes cell proliferation through the RAS-MAPK pathway and inhibits cell death through the phosphatidylinositol 3'-kinase-AKT-mammalian target of rapamycin (mTOR) pathway [8]. Likewise, the HCCR-1 signaling is also known to be regulated by the phosphatidylinositol 3'-kinase-AKT pathway [9].

According to our previous study using a panel of breast cancer cell lines [2], HCCR-1 was highly expressed in breast cancer cell lines with high HER2 overexpression, with some exceptions, that have a mutated p53 and express ER/PR [10-13]. These data indicate that the HCCR-1 overexpression in breast cancer cell lines is well correlated with known breast cancer prognostic markers including high HER2 overexpression [10-13].

Because HER2 overexpression and amplification have important consequences on the prognosis and treatment of breast cancer, their presence must be accurately determined. Currently, two different methods are being used worldwide, immunohistochemistry (IHC) and fluorescence *in situ *hybridization (FISH). These two techniques identify different targets and both have advantages and disadvantages [14]. Which method should be viewed as the gold standard for HER2 determination remains a debate. Recently, other methods have been described as options for HER2 testing. Real time polymerase chain reaction (RT-PCR) [15] and chromogen *in situ *hybridization (CISH) [16] have been proposed as cheaper and easier alternatives to FISH. Acceptable correlations between both methods and FISH have been reported [14]. But the correlations among the various clinical methods of detecting HER2 are imperfect with regard to both prognostication and the prediction of a response to trastuzumab (Herceptin, Genentech).

Multiple studies have demonstrated marked improvement in disease-free and overall survival when trastuzumab is incorporated in therapeutic regimens for HER2 overexpressed tumors [17,18]. Only patients whose tumors strongly overexpress HER2 by immunohistochemistry (IHC) and/or have amplification of the cerb2 gene by FISH, are likely to respond to Herceptin treatment [19].

Testing invasive breast carcinomas for HER2 overexpression/gene amplification has become a standard of practice [20]. Much of the recent literature has focused on the best method for documenting HER2 overexpression (IHC vs. FISH), technical limitations of each method, and the need for consistency and QA in test performance [17,21-23]. The National Comprehensive Cancer Network HER2 Testing in Breast Cancer Task Force concluded that where adequate quality control/quality assurance procedures are followed, either IHC or FISH are acceptable methods [24].

Whether a laboratory uses IHC with FISH for equivocal cases, or uses FISH as the initial HER2 screen, these tests are expensive to perform. Development of a rational approach to selecting cases for HER2 testing would promote cost effectiveness in the health care system [25].

The aim of this study is to analyze the level of HCCR-1 expression in breast cancer tissues in order to demonstrate its correlation to other biomarkers including ER, PR, p53, and HER2, and estimate the possibility of HCCR-1 as a new biomarker candidate for breast cancer.

## Methods

### Tissues and cell lines

Human normal and cancer tissues were obtained during operation. All patients were subjected to the analysis with individual consent for the study. The use of tissue samples was approved by the Ethics Committee of our institution (Kangnam St. Mary's Hospital, Seoul, Korea). Mammalian cell lines were obtained from the American Type Culture Collection (ATCC; Manassas, VA). BT-474, MCF-7 and MDA-MB-231 are human breast carcinoma cell lines from mammary gland. MCF7 and MDA-MB-231 cells express low HER2, and BT-474 cells express high HER2. BT-474 and MCF-7 cells are ER-positive and PR-positive cells. MDA-MB-231 is ER-negative and PR-negative cell line. MCF-7 cells have wild type p53, and MDA-MB-231 and BT-474 cells have mutated p53.

### Northern blot analysis

Total RNA was extracted from fresh human tissues and cell lines using RNeasy total RNA kit (Qiagen). Northern blot analysis was carried out, in which 20 μg of denatured total RNA was electrophoresed on a 1.0% formaldehyde agarose gel and transferred to nylon membrane. Human β-actin cDNA control probe was used as a loading control. All blots were hybridized with the randomly primed [^32^P]-labeled HCCR-1 partial cDNA probe.

### Immunohistochemical analysis and staining interpretation

For immunohistochemistry experiments, paraffin sections (5-μm thick) of normal human breast and cancer tissues were used for stainings using monoclonal antibodies against ER, PR, and p53, and affinity-purified polyclonal anti-HCCR-1 antibodies. Endogenous peroxide and nonspecific binding were blocked by using 3% hydrogen peroxide in TBST and with TBST diluted 1:10 in FBS for 30 minutes, respectively. Then sections were incubated with primary antibodies (diluted in TBST-FBS solution) for 2 hours at room temperature. Corresponding negative controls were incubated in the absence of primary antibody. Aminoethyl carbozole (AEC) was used as the chromogen. After immunostaining, sections were counterstained with hematoxylin. Representative examples of stained tissues were photographed under the microscope. Pathologist counted at least 500 tumor cells in the area most strongly stained for ER, PR and p53. Positive staining for ER, PR, and p53 was defined by nuclear staining. According to most accepted cut-off point, 10% positive staining of tumor cells was used as positive result for ER, PR and p53. Immunohistochemically detected p53 protein is mutated p53 protein due to its prolonged half-life. The positive staining of tumor cells is strongly correlated with p53 mutation [26]. The HER2 immunohistochemical staining results were interpreted according to staining criteria. Positive staining for HER2 was defined by membrane staining. Cytoplasmic staining was not considered positive. Tumor cells showing no immunoreactivity (score 0) or the incomplete and faint membrane staining (score 1+) were considered as negative. HER2 was defined as positive when the complete weak to moderate membrane staining (weakly positive; score 2+) or the complete strong membrane staining (strongly positive; score 3+) was observed in more than 10% of tumor cells. The HCCR-1 immunohistochemical staining results was defined by cytoplasmic staining. The cut-off point of HCCR-1 for positive and negative staining was used 0% of cells. We changed HCCR-1 staining results as positive and negative results instead of stability.

## Results and discussion

### Overexpression of HCCR-1 in breast cancers

**Figure 1:**
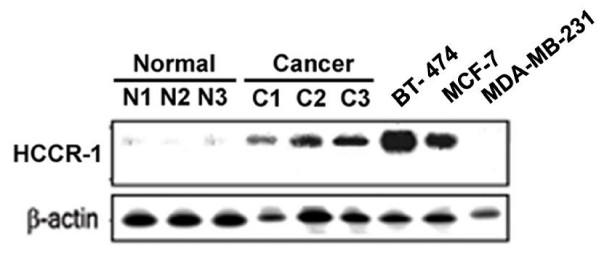
**HCCR-1 expressions in breast cancers**. Northern analyses of HCCR-1 in human breast tissues and cell lines. Comparison of *HCCR-1 *mRNA expressions in breast cancer cell lines (BT-474, MCF-7 and MDA-MB-231), and fresh primary breast cancer tissues (C) and their corresponding normal counterparts (N). Human β-actin cDNA was used as a control probe (bottom panel).

We examined the expression patterns of *HCCR-1 *in human normal tissues, cancer tissues and cell lines. Northern blot analysis revealed an increased expression of *HCCR-1 *in fresh primary human breast cancer tissues compared to their normal counterparts (Fig. 1). HCCR-1 was abundantly detected in BT-474 (ER+/PR+/mutant p53/HER2 3+) and MCF-7 (ER+/PR+/wild p53/HER2 -) cells whereas it was not detected in MDA-MB-231 (ER-/PR-/mutant p53/HER2 -) cells (Fig. 1). The expression level of HCCR-1 was higher in BT-474 than in MCF-7.

### HCCR-1 is well correlated with known breast cancer prognostic parameters

**Figure 2:**
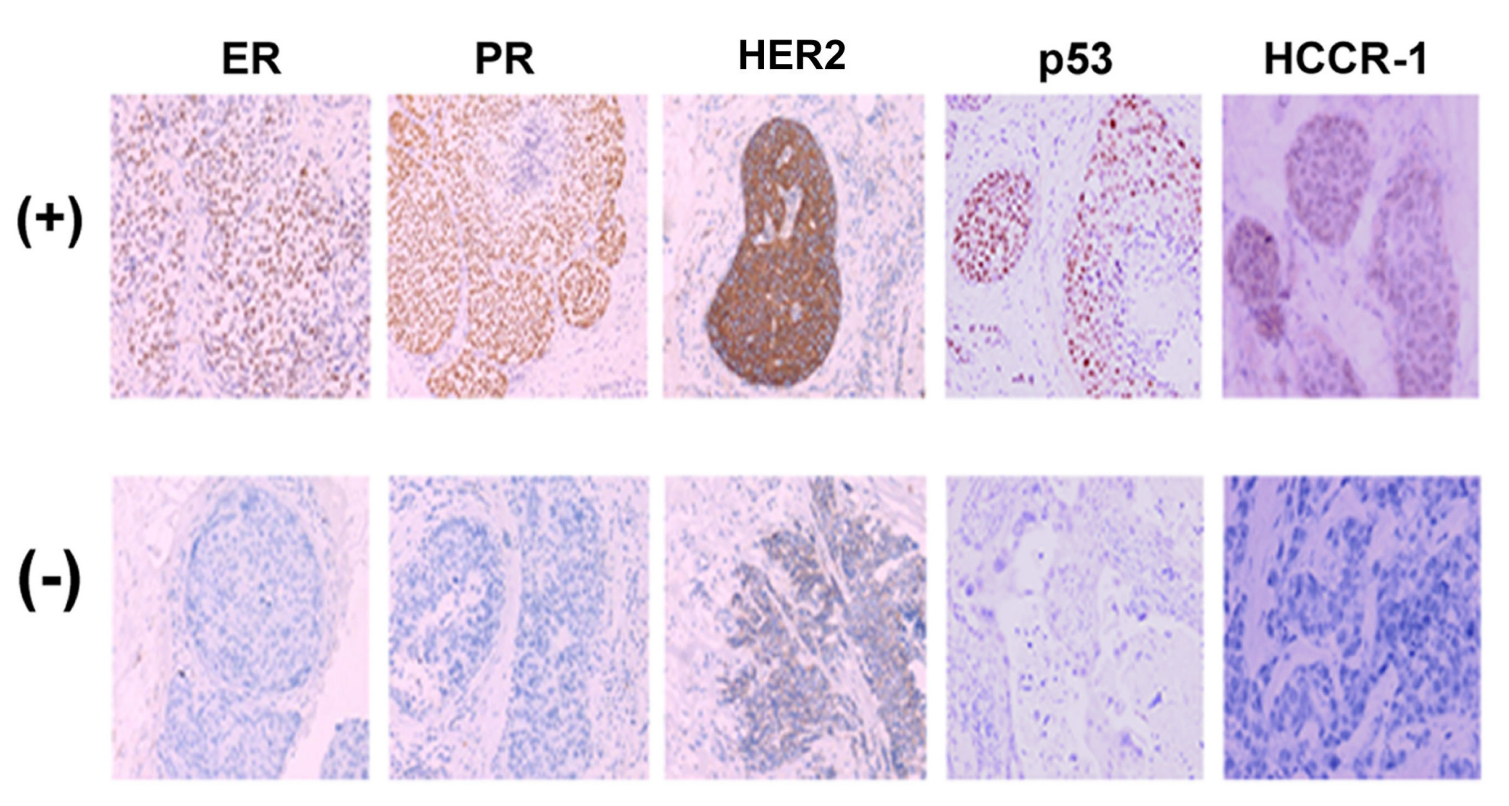
**HCCR-1 is positively well correlated with breast cancer prognostic parameters**. Correlation of HCCR-1 expression levels with known breast cancer prognostic parameters. Immunohistochemical staining for ER, PR, HER2, p53 and HCCR-1 in fresh frozen tissues from human breast cancers. All the breast cancer tissues were derived from pathologically-proven invasive ductal carcinomas. Positive immunostaining for ER, PR, and p53 in the nuclei of carcinoma cells and for HCCR-1 in the cytoplasm of carcinoma cells (top panel). The HER2 immunohistochemical staining showing complete strong membrane staining (3+) in the carcinoma cells (top panel). Negative immunostaining for ER, PR, p53, HER2 and HCCR-1 in breast carcinoma (bottom panel). Original magnification, × 100.

We conducted the following experiment to investigate whether the results of our previous study in which breast cancer cell lines were used [2] can be replicated in this study using fresh primary breast cancer tissues. The expression of HCCR-1 was measured in a panel of fresh 104 primary breast cancer tissues and their normal counterparts (Table 1 and Fig. 2). The increasing expression level of HCCR-1 was observed in the order of breast cancer tissues with (ER+/PR+/mutant p53/HER 3+), (ER+/PR+/wild p53/HER2 2+), and (ER+/PR+/wild p53/HER2 -). HCCR-1 was not detected in breast cancer tissues with (ER-/PR-/p53-/low HER2) (Table 1 and Fig. 2). These results confirm our previous findings in breast cancer cell lines in that HCCR-1 expression is correlated with known prognostic markers for breast cancer.

**Table 1:** Correlation of the HCCR-1 expression levels in 104 primary breast cancer tissues with known breast cancer prognostic parameters including the ER/PR expression, p53 status and HER2 overexpression

*No. of* *Cancer tissues*	*ER* *status*	*PR* *status*	*p53* *status*	*HER2* *Over-expression*	*HCCR-1* *expression*
30	positive	positive	mutant	strong positive	positive
23	positive	positive	wild	moderate positive	positive
23	positive	positive	wild	negative	positive
28	negative	negative	null	negative	negative

All the breast cancer tissues were derived from pathologically-proven invasive ductal carcinomas.

Since HER2 overexpression/amplification has important consequences on the prognosis and treatment of breast cancer [10,13], its presence should be precisely determined. However, the various clinical methods of detecting HER2 are not perfect with regard to both prognostication and the prediction of a response to trastuzumab [14,27-29]. HER2 testing continues to evolve, and many clinical laboratories currently use both tests (IHC and FISH). An efficient testing strategy consists of immunostaining followed by FISH in tumors with 2+ staining intensity [29]. This approach should minimize the risks of not treating patients who might benefit from trastuzumab and of treating patients who are unlikely to have a response; this is a critically important distinction as the use of trastuzumab moves into the adjuvant setting [7]. Reported adjuvant trials used centralized laboratory review because of the high rate of discordant interpretations among individual laboratories [30,31].

Our data reveal that the HCCR-1 expression is tightly associated with HER2 activity in breast cancer tissues. This implies that the prognosis for the breast cancer might be improved when combining the measurement of the HCCR-1 level and the HER2 activity.

## Conclusion

Our results highlight the strong correlation of HCCR-1 expression in breast cancer to other prognostic factors and raise the possibility that HCCR-1 expression can be used as a new prognostic marker for breast cancer.

## Competing interests

The authors declare that they have no competing interests.

## Authors' contributions

SA was the principal investigator of this study. SMS performed the laboratory assays. KHK, SH and HN contributed to the data/sample collection. YSL interpreted staining results. HJK, SMJ and YSL contributed to study design. YJC, SSJ and JWK critically reviewed and wrote the manuscript. All authors read and approved the final manuscript.

## Pre-publication history

The pre-publication history for this paper can be accessed here:

http://www.biomedcentral.com/1471-2407/9/51/prepub
